# Anomalous dielectric behaviors of electrolyte solutions confined in graphene oxide nanochannels

**DOI:** 10.1038/s41598-021-98326-9

**Published:** 2021-09-21

**Authors:** Bo Hu, Haochen Zhu

**Affiliations:** 1 State Key Laboratory of Pollution Control and Resources Reuse, Key Laboratory of Yangtze River Water Environment, Ministry of Education, College of Environmental Science and Engineering, Tongji University, 1239 Siping Rd., Shanghai, 200092 People’s Republic of China; 2 Shanghai Institute of Pollution Control and Ecological Security, Shanghai, 200092 People’s Republic of China

**Keywords:** Chemical physics, Information theory and computation, Statistical physics, thermodynamics and nonlinear dynamics

## Abstract

Dielectric behavior of salt aqueous solutions with various concentration in pristine and oxide graphene nanochannels has been investigated by means of molecular dynamic simulations. The motivation in performing this integrated set of simulations was to provide deep insight into the interaction between the size of the enclosure and the oxidation degree of the membrane sheets on the dielectric properties. It was shown that the dielectric permittivity of both aqueous and NaCl solution in confined phase exhibits an anisotropic behavior. The in-plane component decreases with the increase of the concentration of NaCl solution while an increase of the out-of-plane dielectric is observed and these out-of-plane components exhibit a non-monotonous trend and thus exist a critical concentration of NaCl solution with 0.2 mol/L and 0.4 mol/L for both pristine and oxide graphene nanochannels, respectively. This peculiar dielectric behavior results from the addition of ions that significantly perturb the hydrogen bonding network of the confined system, and hence leading to a fluctuation of dipolar of water molecules and dielectric permittivity.

## Introduction

With the increasing of world population and the rapid development of urbanization and industrialization, water resources are seriously polluted and the available fresh water is reduced. Although about 70% of the earth’s surface is covered by water, the amount of fresh water available for direct use is only a fraction of that. Membrane desalination technology, such as reverse osmosis, has been more prevalent for the separation of salt ions in marine water due to its advantages of high efficiency and simple operation^1–4^. However, some defects such as high energy consumption, low water permeance and self-fouling limit its potential for wide application. Therefore, the design of new desalination materials that are safe, environmental and cost-efficient is extremely necessary in the field of materials science and water treatment process. Thanks to the great improvements in the performance of materials science in recent years, a large number of nanomaterials are used to ameliorate the water purification process^5–7^. Notably, laminated graphene oxide (GO) membranes are considered as one of the materials with great promise for seawater desalination due to their excellent ionic filtration performance, rapid transport of water molecules and richly functional groups on the graphene sheets. Nowadays, a large amount of theorical and experimental work have focused on the pressure-driven flow behavior of pure water and salty water through the GO nanochannel. Liu et al.^8^ investigated the influences of the channel interval and the pressure difference on the water flow inside bi-layer GO nanochannel by molecular dynamics (MD) simulations and they found that the flow of water increased monotonously with a rise of the channel space and driving force. Subsequently, they studied the effect of external electric field on the salt rejection performance. Results showed that ions could be captured in nanochannel and could ameliorate permeable capability and channel fouling without obliterating permeation flux as a perpendicular electric field is implemented^9^. Moreover, Kim et al.^10^ noticed that water flow rate enhanced with the increase of the driving force and an improvement of ion repulsion as increasing the oxidation rate on nanoporous graphene filters. More recently, Giri et al.^11^ investigated both the oxidation rate and the layer spacing on the salt ion retention performance in GO nanochannels, observing a trade-off between the good desalination ability inside the narrower channel and the higher water permeability inside the wider channel. However, due to the complexity of separation mechanism caused by the change of physical properties at the nanoscale, the application of graphene-based membranes is still limited compared to its potential competence. Therefore, comprehending of physical behavior of water and salt solutions under nanoconfined systems is of great significance in water desalination processes. Among which, the dielectric permittivity (ε) is playing the vital role in governing the liquid medium transfer features with a confined system. For example, dielectric behavior straight regulates the nanofiltration, reverse osmosis, and nanofluidic processes in application of desalination. Thus, it is considerable to evaluate the dielectric permittivity of confined aqueous and saline solution since the shortage of awareness of dielectric characteristics forcefully limits the capacity to model solvent- substrate interactions and, in particular, restricts our comprehension of the behavior of water and electrolyte fluids under confinement. To this end, we reported recently a tortuous dielectric behavior of pure water inside the GO nanochannel with various oxidation degrees. Results indicated that the degree of oxidation on the graphene sheets had a great influence on the dielectric permittivity of confined water and the dielectric properties exhibited a non-monotonous trend with the increase of oxidation rate^12,13^.

Electrolytes solution are considered as sophisticated systems and thus it is extremely difficult to understand the dynamic construction. Due to the electrostatic and steric effects of ions, the original structure of the water may change, forming the solvated shell of ions under the induction of these effects. These configurational alterations lead to complex kinetics of hydrogen bonds, water- substrate and the formation of ion pairs. In fact, the dielectric permittivity of salt solution decreases with the increase of electrolyte concentration, which has been observed by Drude and Nernst for about one century^14^. During this period, a great deal of effort has been devoted to understanding this physical phenomenon^15–22^ and most of them were elucidated in the light of dielectric saturation^18,20^. This phenomenon is caused by a relatively strong electric field, leading to the shielding of water dipoles and thus an orientation of water dipoles around the ions. This latter therefore results in a reduction of dielectric permittivity with increasing the concentration of electrolyte solution. However, another competitive phenomenon that affects the dielectric permittivity occurs due to the spoilage of the hydrogen bond (HB) network and the reduction of the interspace when salt ions are present in an aqueous solution^18^. This destruction HB network is conducive to the contribution of entropy, thus increasing the dipole fluctuation and dielectric permittivity of water. On the contrary, like pure water confined in a nanometer-sized system, the dynamical and physical properties of saline water under nanometer dimension may change differently from that of bulk phase. Although numerous works have focused their attention on nano-structured applications such as desalination by nanoscale membranes, the dielectric properties of salt solution in confined phase are less investigated. Even so, Zhu et al.^21^ reported an abnormal dielectric behavior of saline water in a hydrophilic silica nanopore by molecular dynamics (MD) simulations. Subsequently, Renou et al.^19^ observed the different dielectric regimes as a function of salt concentration in a nanocavity. More recently, Jalali et al. reported the impact of electrolyte solution on the dielectric properties inside graphene bilayers. However, the response of dielectric permittivity of saline water in graphene oxide membranes has hardly been investigated.

In the present work, we perform a sequence of MD simulations of aqueous sodium chloride solution confined in a nanochannel formed by either pristine graphene (PG) or graphene oxide sheets with the same interval space. The influence of the concentration of NaCl solution on both PG and GO bilayers has also been investigated. The results are provided in prospect of building up a fundamental understanding of micro-level mechanisms in the field of desalination, with the extended target of facilitating the progress of new and urgently needed technologies to cope with the increasing demand for plain water.

## Models and simulation details

**Figure 1:**
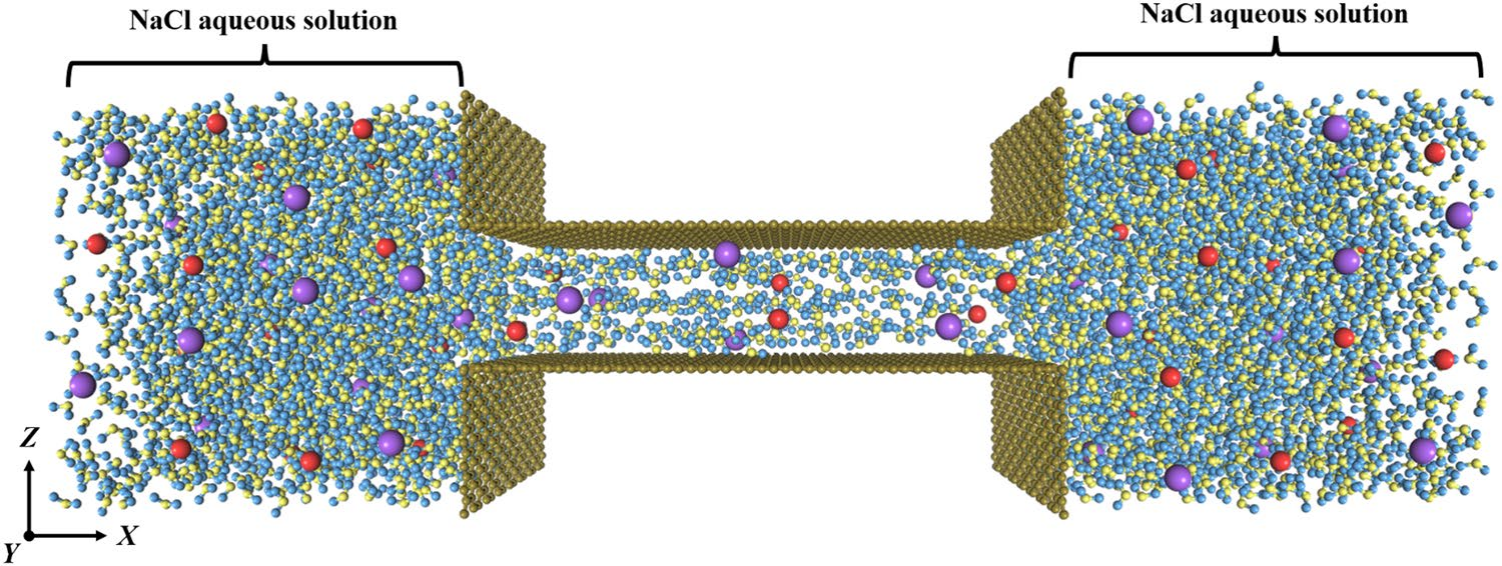
Perspective projection of the simulated nanochannel consisted of graphene bilayers and two reservoirs of NaCl aqueous solution at both ends. Dark green color corresponds to the graphene sheets, blue the hydrogen, and yellow the oxygen atoms. Red and violet colors indicate the Na^+^ and Cl^−^, respectively.

The investigated system is composed of a horizontal nanochannel constituted by a graphene sheet overlapped on top of another one in parallel with the interlamellar spacing of d = 1.2 nm. Two reservoirs of NaCl solution are initially situated to both ends of the nanochannel with the same dimension of 40 × 36 × 36 Å^3^ in *x*, *y* and *z* direction, respectively (see Fig. 1). The graphene nanochannels in the center of the two saline reservoirs have a constant length *L* 70 Å. Four restrained PG walls are placed perpendicular to the graphene plane to limit the movement of water and ions into the horizontal nanochannel. The nanochannels formed by GO nanosheets are performed by the addition of hydroxyl groups distributed randomly on the graphene sheets with a lower oxidation degree defined as follow: ${\text{D}}_{o} = n_{{{\text{OH}}}} /n_{c} = 5\%$ (where $n_{{{\text{OH}}}}$ and $n_{c}$ are the number of hydroxyl groups and carbon atoms, respectively). The effect of salt concentration has also been addressed by considering NaCl solutions at different concentrations (i.e. $c_{{{\text{Nacl}}}} =$ 0.1 mol/L, 0.2 mol/L, 0.3 mol/L, 0.4 mol/L, 0.5 mol/L, 0.6 mol/L, 0.8 mol/L and 1.0 mol/L).

All atomic/molecular simulations are executed applying the Large-scale Atomic/Molecular Massively Parallel Simulator (LAMMPS) package^23^. The bonded and non-bonded interatomic interactions of GO are modelled by CHARMM27 force field^24^. For liquid/vapor systems, it has been well established that the polarizability of water molecules does not affect the ion density situated in the region of solution-matrix interfacial^25^. Thereby, we used in this work the non-polarizable water model TIP4P/2005 which has been considered to provide a puissant capability for multiple physical properties. This model is a rigid model based on the Bernal-Fowler geometry and functionality and reproduces most properties of bulk water under ambient conditions^26^. For sodium and chloride ions, we use an unpolarizable force field^27^ since the polarizability has an inappreciable effect on the dynamics and dielectric properties^19,21,28^. Particle–particle–particle–mesh (PPPM) method are applied to calculate the long-range Coulombic with a cut of 12 Å and vdW interactions are modelled applying the 12–6 Lennard–Jones (LJ) potential truncated at 10 Å via the Lorentz–Berthelot combination rule. Table 1 gives the force field parameters and charges of each atom used in the simulations. All the molecular dynamics simulations are carried out in the NVT ensemble using Nosé-hoover thermostat^29,30^ with the periodic boundary conditions at 298 k and 1 bar. The graphene matrix of nanochannel is kept rigid while the structure of water molecule is restrained applying the SHAKE algorithm^31^. The equation of motion are resolved by employing the velocity Verlet algorithm^32^ with a time interval of 1 fs. Initially, simulation is performed for a long run of 2 ns with a timestep of 1 fs to ensure equilibration of the system. Then, another 10 ns of simulation has been carried out with a timestep of 1 fs to accomplish the dada analysis and acquisition.

**Table 1:** LJ parameters charges of atoms in the simulation.

Atom	*σ* (Å)	*ε* (kcal mol^−1^)	Charge (*q*_*e*_)
*O*_*w*_ (oxygen of water)	3.1589	0.7749	0
*H*_*w*_ (hydrogen of water)	0.000	0.000	0.5564
*O*_*h*_ (oxygen of hydroxyl)	3.070	0.170	− 0.585
*H*_*h*_ (hydrogen of hydroxyl)	0.000	0.000	0.435
C (C–C)	3.851	0.105	0.000
C (bonded to hydroxyl)	3.550	0.070	0.150
Na^+^	4.070	0.710	1.000
Cl^−^	4.020	0.001	− 1.000

## Space-dependent dielectric permittivity calculation

The dielectric permittivity of NaCl solution confined in graphene bilayers presents an anisotropic characteristic, which ε varies with the position of the media and turns into a second-rank tensor. It is necessary thus to grasp precise information of the space-dependent dielectric properties since they rule the mass transfer and retention properties in many separation processes such as nanofiltration. Obviously, it is difficult to consider the global dielectric permittivity under graphene bilayers confinement.

In the present graphene bilayer planar system, the non- homogeneity is along the *z*-direction. The characterist along the *x* and *y* directions results in an isotropic electric field $E_{x} = E_{y}$. According to the Maxwell’s law ($\nabla { } \wedge { }{\mathbf{E}}\left( z \right) = 0$), the physical properties in the *z*-direction contain

1 $$\frac{{\partial E_{y} \left( z \right)}}{\partial z} = \frac{{\partial E_{x} \left( z \right)}}{\partial z} = 0 \to E_{y} \left( z \right) = E_{x} \left( z \right) = E_{\parallel } \;{\text{and}}\;E_{z} \left( z \right) = E_{ \bot } \left( z \right)$$

where $E_{\parallel }$ and $E_{ \bot }$ represents the in-plane and out-of-plane electric field. It should be note that $E_{z} \left( z \right)$ is undeclared according the Maxwell’s law. The space-dependent dielectric tensor thus can be written by both parallel and orthogonal to the graphene surfaces

2 $$\upvarepsilon \left( z \right) = \left( {\begin{array}{*{20}c} {\varepsilon_{\parallel } \left( z \right)} & 0 & 0 \\ 0 & {\varepsilon_{\parallel } \left( z \right)} & 0 \\ 0 & 0 & {\varepsilon_{ \bot } \left( z \right)} \\ \end{array} } \right)$$

where $\varepsilon_{\parallel }$ and $\varepsilon_{ \bot }$ denotes the in-plane and out-of-plane dielectric, respectively. According to the theoretical frame proposed by Bonthuis et al.^33^, the local displacement field can be defined by the electric field and written as,

3 $$\Delta D_{\parallel } \left( z \right) = \varepsilon_{0} E_{\parallel } + \Delta m_{\parallel } \left( z \right) = \varepsilon_{0} \upvarepsilon_{\parallel } \left( z \right)E_{\parallel } .$$

Then, the in-plane dielectric permittivity can be defined in Eq. (4),

4 $$\upvarepsilon_{\parallel } \left( z \right) = 1 + \frac{{\Delta {\text{m}}_{\parallel } \left( z \right)}}{{\varepsilon_{0} E_{\parallel } }}$$

where $\varepsilon_{0}$ is the vacuum dielectric constant, $\Delta {\text{m}}_{\parallel } \left( z \right)$ is the polarization density and calculated from the linearized version of the fluctuation dielectric correlations function^34^,

5 $$\Delta {\text{m}}_{\parallel } \left( z \right) = \beta \left[ {\left\langle {{\text{m}}_{\parallel } \left( z \right){\text{M}}_{\parallel } } \right\rangle_{0} - \left\langle {{\text{m}}_{\parallel } \left( z \right)} \right\rangle_{0} \left\langle {{\text{M}}_{\parallel } } \right\rangle_{0} } \right]F_{\parallel }$$

where $\left\langle \cdots \right\rangle_{0}$ represents a ensemble averages over the diverse configurations, $\beta$ denotes the inverse thermal energy, ${\text{M}}_{\parallel }$ is the parallel dipole moment of the system, $F_{\parallel }$ represents an external parallel homogeneous electrical field and refers to ${ }E_{\parallel }$. Considering both Eqs. (4) and (5), we can thus acquire the evaluation expression of in-plane dielectric permittivity as follow,

6 $$\upvarepsilon_{\parallel } \left( z \right) = 1 + \beta \varepsilon_{0}^{ - 1} \left[ {\left\langle {{\text{m}}_{\parallel } \left( z \right){\text{M}}_{\parallel } } \right\rangle_{0} - \left\langle {{\text{m}}_{\parallel } \left( z \right)} \right\rangle_{0} \left\langle {{\text{M}}_{\parallel } } \right\rangle_{0} } \right].$$

The out-of-plane component $\varepsilon_{ \bot } \left( z \right)$ can be obtained by combining another Maxwell’s equation $\nabla \cdot {\mathbf{D}}\left( z \right) = 0$ and the inverse dielectric response ${\mathbf{E}}\left( {\mathbf{r}} \right) = \frac{{\mathbf{D}}}{{\varepsilon_{0} \varepsilon_{r} \left( r \right)}}$. We have thus the homogeneous out-of-plane displacement,

7 $$\Delta D_{ \bot } \left( z \right) = \varepsilon_{0} E_{ \bot } + \Delta {\text{m}}_{ \bot } \left( z \right).$$

Combining the inverse dielectric response and Eq. (7) gives,

8 $$\upvarepsilon_{ \bot } \left( z \right)^{ - 1} = 1 - \beta \varepsilon_{0}^{ - 1} \left[ {\left\langle {{\text{m}}_{ \bot } \left( z \right){\text{M}}_{ \bot } } \right\rangle_{0} - \left\langle {{\text{m}}_{ \bot } \left( z \right)} \right\rangle_{0} \left\langle {{\text{M}}_{ \bot } } \right\rangle_{0} } \right]$$

where ${\text{M}}_{ \bot }$ is the orthogonal dipole moment of the system. To calculated the space-dependent dielectric components, we have accounted the dipole moments of water molecules and the dipolar relaxation of ions pair. Indeed, at higher concentration ions pair can be form and act as a dipole and then to contribute the dielectric constant. Thus from the computation of out-of-plane distribution function (RDF) between Na^+^ and Cl^−^ we have determined a separated distance of 3.2 Å. Afterwards this characteristic distance has been used to compute the whole of the ions pairs and the dipolar relaxation are then accounted in the dielectric constant calculation.

## Results and discussion

**Figure 2:**
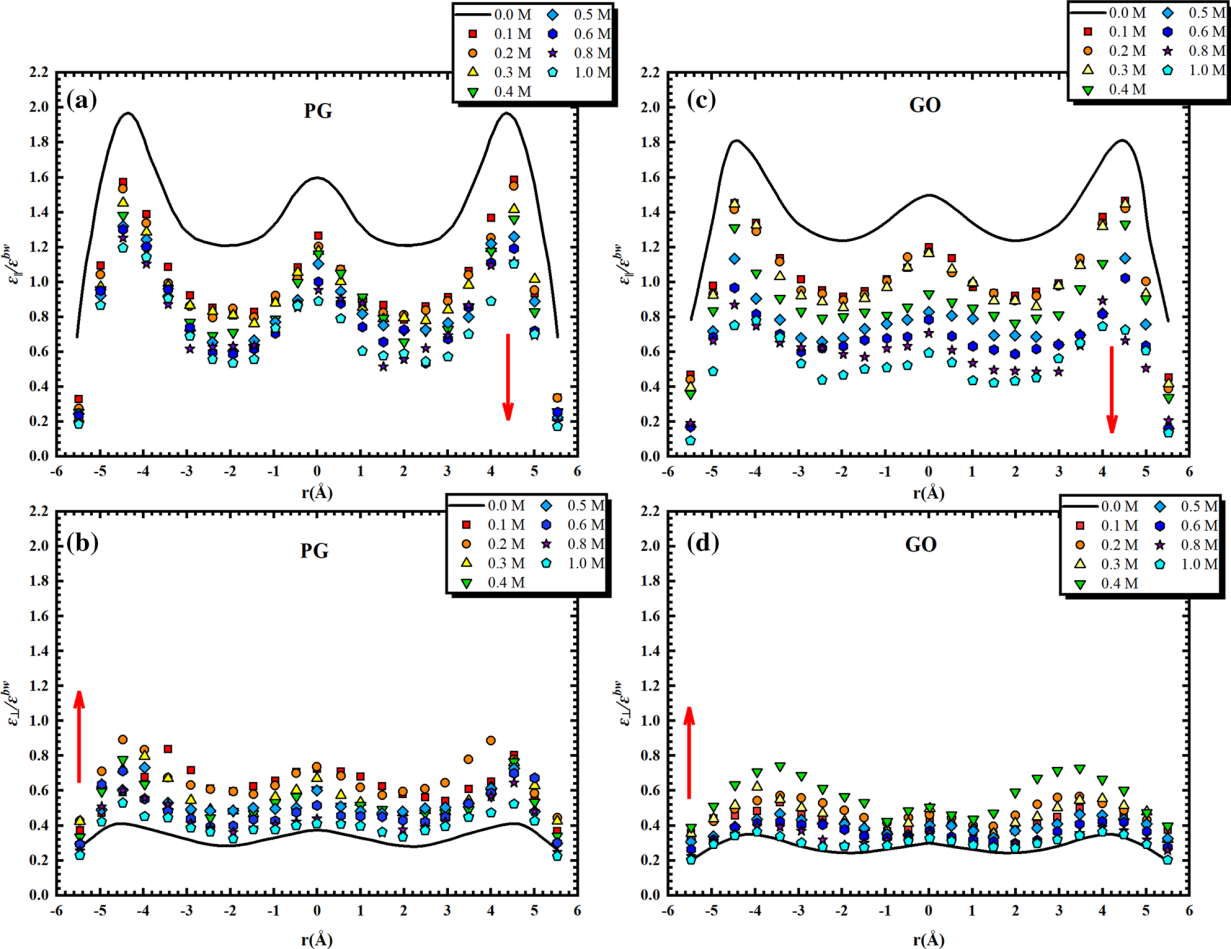
Ratio of out-of-plane ($\varepsilon_{ \bot } )$ and in-plane ($\varepsilon_{\parallel }$) dielectric components of various salt concentrations in both PG (**a**, **b**) and GO (**c**, **d**) channels to that of water in bulk phase ($\varepsilon^{bw}$).

As introduced in Part 3, the dielectric permittivity of NaCl solution confined in graphene bilayers varies with the position of the media and becomes a second-rank tensor. Figure 2a–d reports the ratio of out-of-plane $\left( {\varepsilon_{ \bot } } \right)$ and in-plane ($\varepsilon_{\parallel }$) dielectric components of various salt concentrations in both PG ((a) and (b)) and GO ((c) and (d)) channels to that of water in bulk phase ($\varepsilon^{bw}$). It is worth mentioning that we describe the dielectric properties in a relative way since the value of dielectric permittivity for bulk water devised by TIP4P/2005 model ($\varepsilon^{bw} = 60$) is smaller than the laboratorial one ($\varepsilon^{bw} = 78$). Hence, this manner can more precisely investigate the dielectric behaviours in a confined phase. As shown in Fig. 2a–d, the in-plane component $\varepsilon_{\parallel }$ for both PG and GO cases decreases with the increase of the concentration of NaCl solution while an increase of the out-of-plane dielectric $\varepsilon_{ \bot }$ is observed.

**Figure 3:**
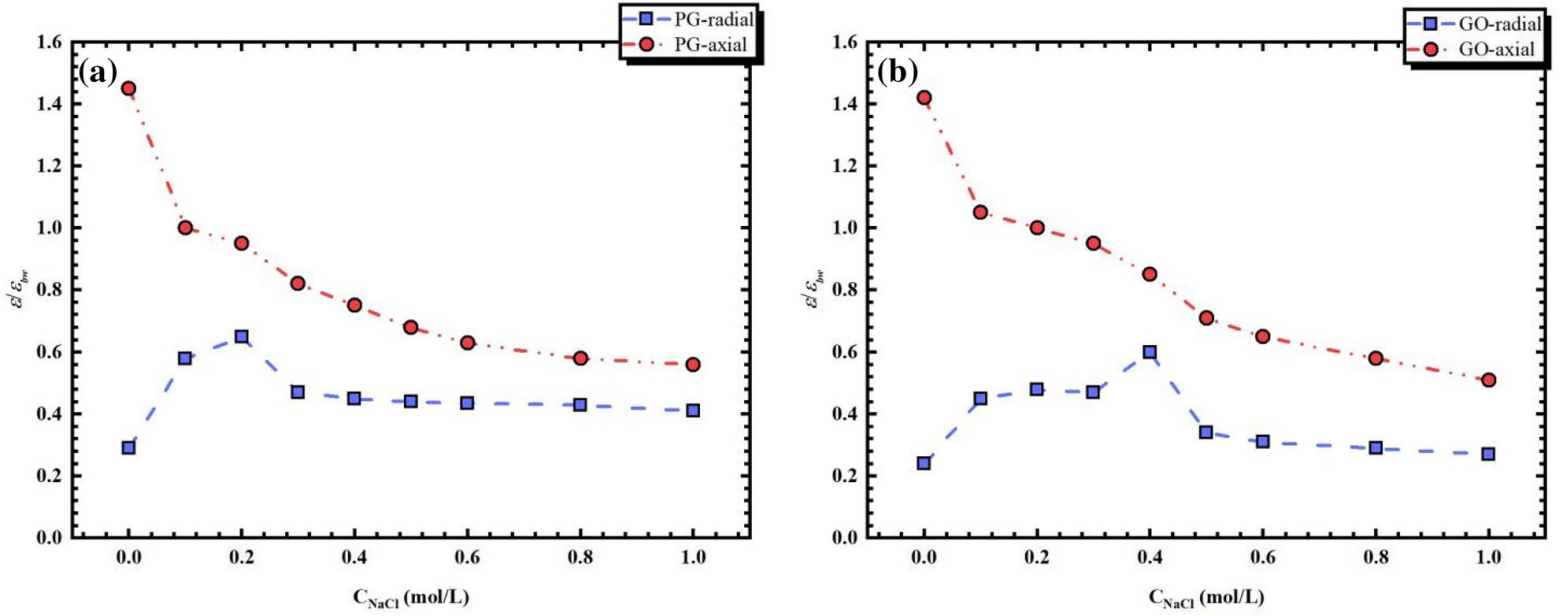
Dielectric permittivity of NaCl solution in both PG and GO graphene bilayers as a function of salt concentrations.

**Figure 4:**
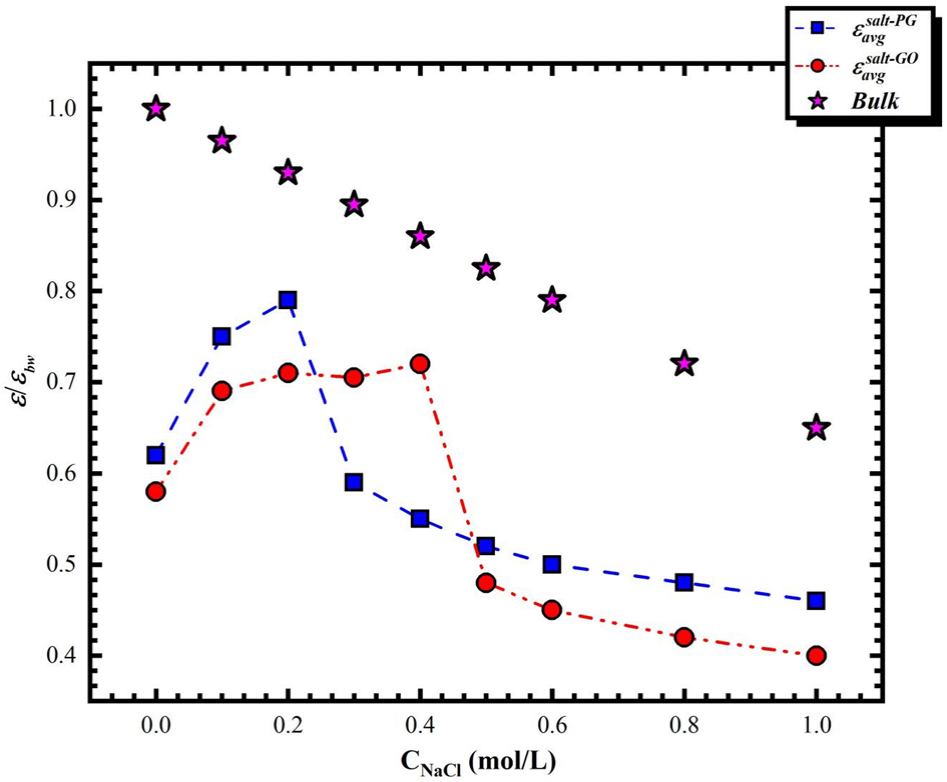
Average dielectric permittivity of confined NaCl solution ($\varepsilon_{avg}^{salt}$) and that of in bulk phase (pentagram symbol) as a function of ${\text{c}}_{{{\text{NaCl}}}}$.

In order to further comprehend the dielectric behaviors in PG and GO channels for NaCl solution, we report in Fig. 3 the dielectric permittivity of NaCl solution in both PG and GO graphene bilayers as a function of salt concentrations. As shown in Fig. 3 the dielectric permittivity of both aqueous and NaCl solution in confined phase exhibits an anisotropic behavior. For pure water in confined situation, we observe a reduction of $\varepsilon_{ \bot }^{{}}$ and an augmentation of $\varepsilon_{\parallel }^{{}}$ relative to the bulk phase. For NaCl solution, $\varepsilon_{\parallel }^{salt}$ decreases with the increase of NaCl concentration in both PG and GO nanochannels, which is consistent with the trend of permittivity of NaCl solution in bulk phase (pentagram symbol in Fig. 4). Indeed, the reduction in permittivity of water with a raising of salt concentration is well known and has been measured experimentally since one century^17,20^. However, the out-of-plane components for both PG and GO bilayers exhibit a non-monotonous trend and thus exist a critical concentration of NaCl solution with 0.2 mol/L and 0.4 mol/L for PG and GO channels, respectively. Similar findings have been observed in our previous molecular dynamics simulations with a cylindrical silica nanopore^21^. The out-of-plane restructuring and the diverse concentration dependence of the permittivity characteristics of NaCl solutions in confined PG and GO channels exhibit an unusual microscopic framework of water molecules. The re-organization of water will be further discussed by the dipolar fluctuations and hydrogen bonds (HBs). In addition, in order to compare the behaviors of the permittivity in PG and GO channels more intuitively, Fig. 4 presents the overall average dielectric permittivity of confined NaCl solution ($\varepsilon_{avg}^{salt}$) and that of in bulk phase (pentagram symbol) as a function of ${\text{c}}_{{{\text{NaCl}}}}$. The method to calculate this average value can be found elsewhere^13^. The dielectric permittivity of salt solutions confined in both nanochannels are smaller than that in bulk medium, which is mainly due to the fact that the confining effect that results in the reduction of water competence to respond to the external electric field and thus the dielectric permittivity. Comparing the dielectric permittivity of salt solutions inside PG and GO channels, we observe that both of them behave similarly to their out-of-plane components (i.e. a non-monotonous variation of dielectric).

In PG nanochannel, the dielectric permittivity increases with the raising of salt concentration and reaches its maximum value when $c_{{{\text{Nacl}}}} =$ 0.2 mol/L. The rise in the dielectric of NaCl solution with relative low concentration can be attributed to the re-orientation of water molecules to maintain the HB network. Indeed, although the addition of salt ions can destroy the original HB network, the low ion concentration allows enough water molecules to recombine to support the HB network. Therefore, the fluctuation of dipole moment caused by the recombination of water molecules leads to the increase of dielectric constant. As $c_{{{\text{Nacl}}}} >$ 0.2 mol/L, the interaction between water and ions becomes predominant and the HB network is poignantly impacted. In fact, at higher concentration there is a relatively strong electric field around the ions in the solution and the dipolarized solvent molecules are thus oriented on its surface. This phenomenon is so-called dielectric saturation effect and plays a decisive role. As a result, adjacent water molecules are no longer able to support an intact HB network with the circumambient fluid. A raising proportion of the hydrogen bond probabilities may lose near to the pairs of ions that can be not taken place at $c_{{{\text{Nacl}}}} \le$ 0.2 mol/L. The losing HB is equilibrated by a favorite orientation of water dipoles around ions.

For NaCl solution confined in GO nanochannel, similar trend of dielectric permittivity has been observed with the increasing of salt concentration. However, the highest permittivity is not achieved abruptly and shifted to a higher $c_{{{\text{Nacl}}}}$ with respect to the PG case. As shown in Fig. 4, the dielectric permittivity of NaCl solution inside GO channel undergoes three characteristic stages with (i) 0.0 mol/L to 0.1 where dielectric constant increases abruptly, (ii) a dielectric plateau from 0.1 mol/L to the critical 0.4 mol/L with the highest $\varepsilon_{avg}^{c - GO}$, and (iii) a decreasing region of dielectric constant with increasing the salt concentration. The increase of dielectric with $c_{{{\text{Nacl}}}} = 0.1$ mol/L may firstly attribute to the re-orientation of water molecules to maintain the HB network just like the situation in PG case. It is worth mentioned that our GO nanosheets are randomly distributed with about 5% hydroxyl groups, which may form the water- substrate electrostatic interaction that serves on a saturation field for aqueous solution and leads to a decrease of dielectric permittivity^19,21^. However, this interaction is gradually shielded by the addition of ions with a low concentration, which is in favor of an increase of $\varepsilon_{avg}^{c - GO}$. With the increase of the salt concentration ($c_{{{\text{Nacl}}}} = 0.2\sim 0.4$ mol/L), the effect of dielectric saturation emerges gradually and forms a competitive aspect with the water- substrate electrostatic interaction. This latter is mainly caused by the presence of hydroxyl groups on the membrane surface and is verified by another MD simulation with a higher oxidation degree (${\text{D}}_{o} = 30\%$), which shows an appreciable increase of the critical NaCl concentration. When $c_{{{\text{Nacl}}}} > 0.4$ mol/L, water-substrate electrostatic effect close to the membrane surface is completely shielded by salt ions and the effect of dielectric saturation prevails.

Comparing the permittivity of NaCl solution in the two nanochannels, we observe that the dielectric permittivity in GO channel is smaller than that in PG channel due to the water- substrate electrostatic effect when $c_{{{\text{Nacl}}}} < 0.2$ mol/L. However, this trend is broken when $0.2\;{\text{mol}}/{\text{L}} < c_{{{\text{Nacl}}}} \le 0.4\;{\text{mol}}/{\text{L}}$. As discussed above, the further increase of salt concentration leads to the precipitous decline of dielectric in PG channel due to the dielectric saturation while a dielectric plateau is formed in GO case, resulting from the competition between the dielectric saturation and water-substrate electrostatic interaction. For higher salt concentrations ($c_{{{\text{Nacl}}}} > 0.4\;{\text{mol}}/{\text{L}}$), although the predominance of dielectric saturation results in a rapid decrease of the permittivity for both PG and GO nanochannels, a relative low dielectric constant in GO channel is presented with respect to that in PG one. This is mainly because the addition of hydroxyl groups on the membrane surface compress the space of the channel, which further limits the rotation of water molecules and thus leads to the decline of the dielectric permittivity. The above analysis of the effect of salt solution concentration on dielectric constant in both PG and GO channels accords with our previous work in a silica nanopore, which both systems possess similar hydrophilic surface^21^. The difference between these two systems is the curvature of membranes.

**Figure 5:**
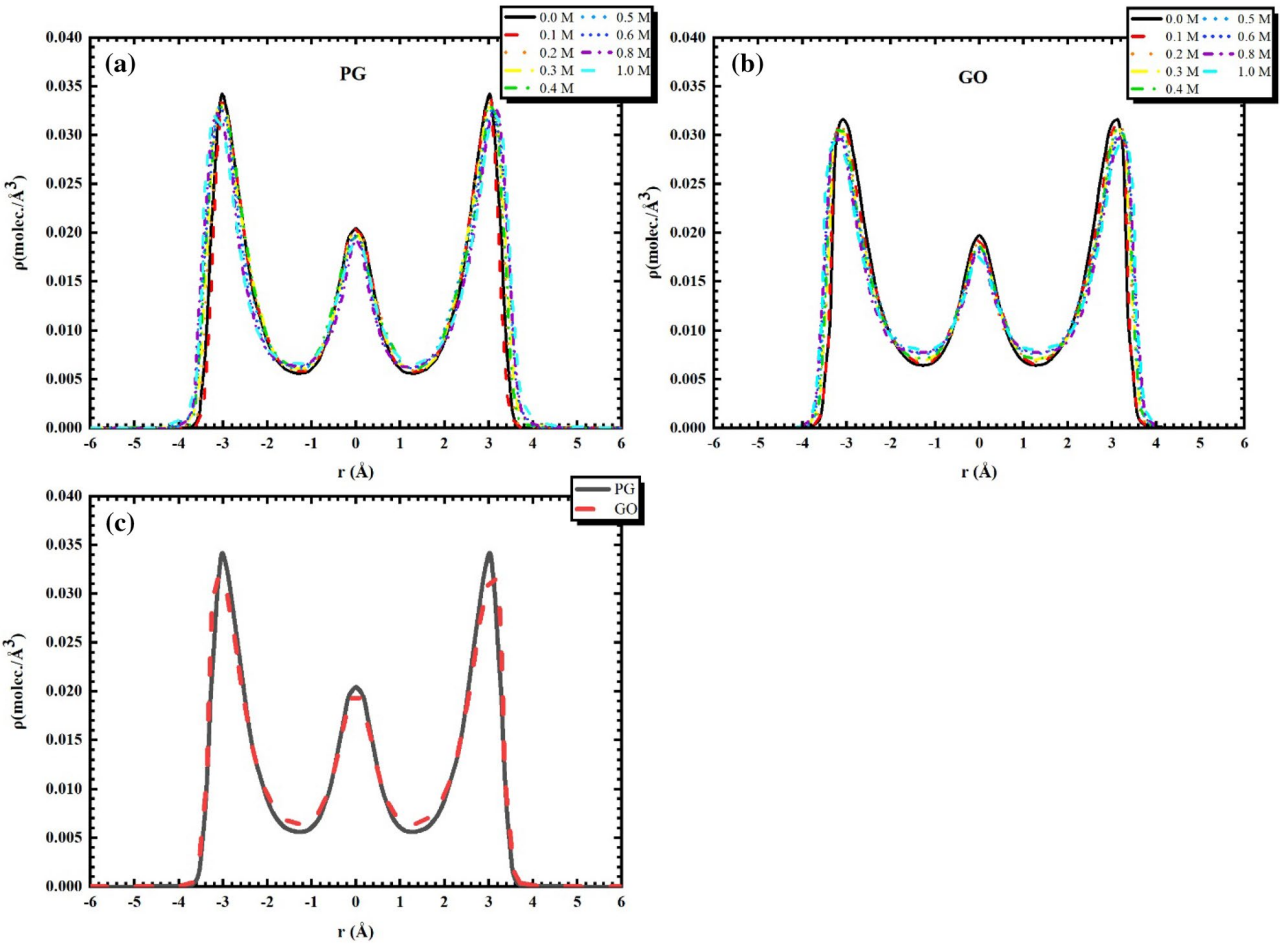
Out-of-plane density of water with various NaCl concentrations inside the (**a**) PG and (**b**) GO nanochannels. (**c**) the comparation of pure water density for the two types of channels.

In order to probe into the relevance between the distribution of water and the permittivity, Fig. 5a–c plot the out-of-plane density of water with various NaCl concentrations for both PG and GO nanochannels. Three water layers form in the central of the channel and near the two interfacial regions with a relatively high density and the local density of water is scarcely influenced by the existence of ions regardless of the $c_{{{\text{Nacl}}}}$. Similar results of water density profiles with various salt concentrations were found in a silicate nanocavity investigated by Ghoufi et al^19^. Actually, the distribution and density of pure water inside PG and GO bilayers are slightly different (see Fig. 5c), which may transform with the variation of oxidation degree on the membrane surface. Generally speaking, the higher the degree of oxidation, the lower the peak of water density presents. Such structure variation of water molecules inside the graphene bilayers is a general feature and has been fundamentally discussed in our previous work^12,13^. Obviously, we cannot directly obtain the explications of dielectric properties reported in Fig. 4 for PG and GO channels since the oxidation rate of graphene is only 5%.

**Figure 6:**
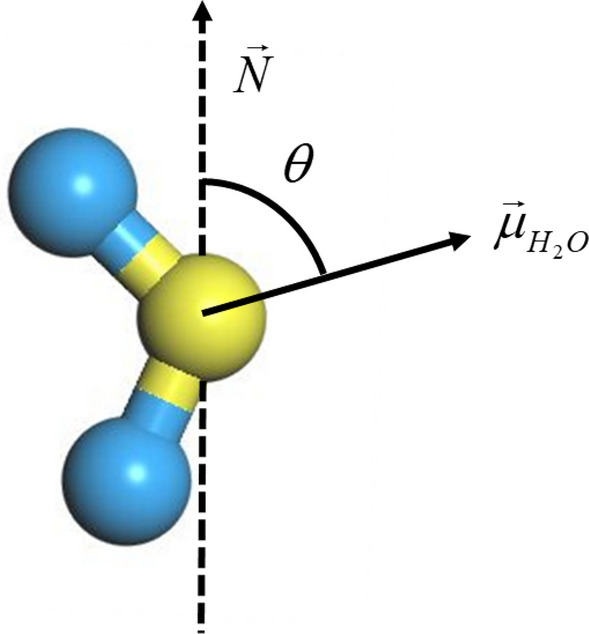
Definition of angles $\theta$ between the vectors of water dipole moment $\vec{r}_{{\mu_{{{\text{H}}_{2} {\text{O}}}} }}$ and the normal to the bilayers surface $\vec{r}$.

**Figure 7:**
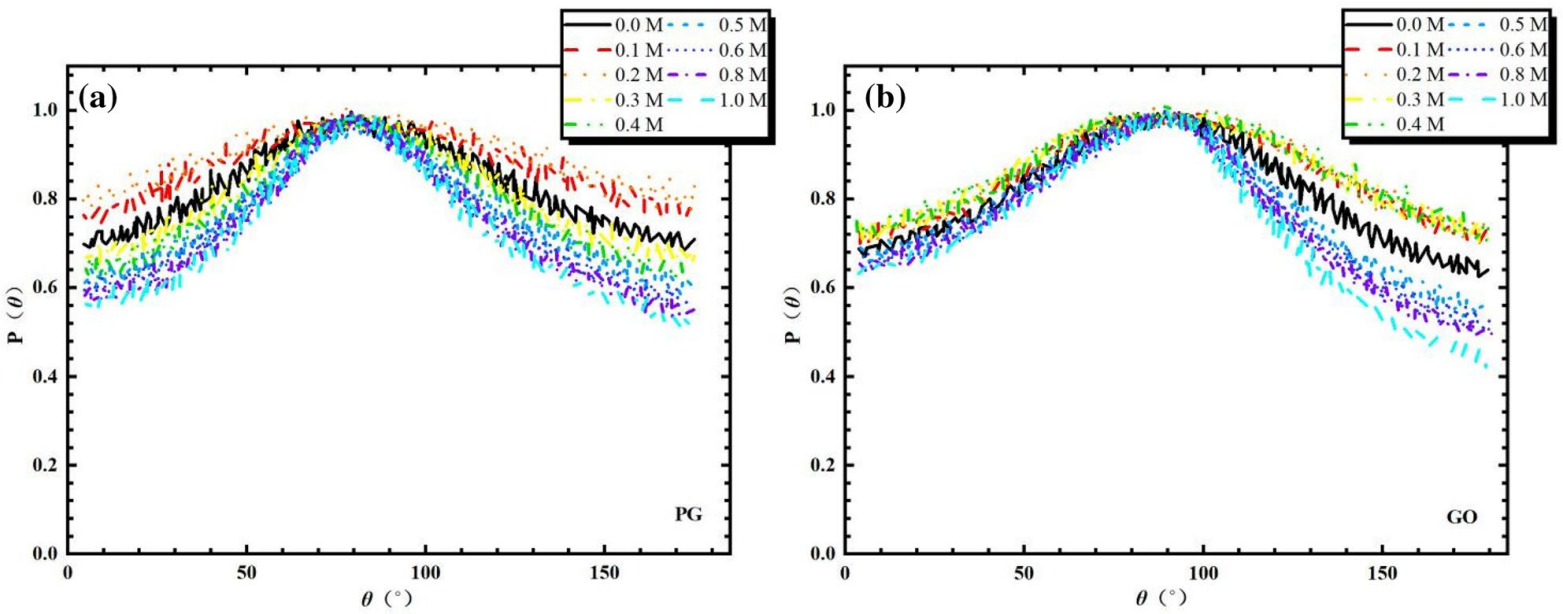
Probabilities of angle distributions $\theta$ for different concentration of NaCl solution in both PG (**a**) and GO channels (**b**).

According to the calculation of dielectric permittivity introduced in Part 3, ε is a metrics of the competence of water molecules to adapt themselves to respond to the appearance of an electric field. As a result, the permittivity is straightly influenced by the orientation of water molecules. In the nanochannel, the permittivity behavior of water is linked to the orientation of water dipoles in relation to the graphene surface. Based on this, we can analyze the structure of water molecules applying the angle θ that regulated on base of the Cartesian coordinate for molecular and defined between the vectors of water dipole moment $\vec{r}_{{\mu_{{{\text{H}}_{2} {\text{O}}}} }}$ and the normal to the bilayers surface $\vec{r}$, as illustrated in Fig. 6a. We show in Fig. 7a,b the probabilities of angle distributions for different concentration of NaCl solution in both PG and GO channels. For the case of PG channel (see Fig. 7a), a peak of angular distributions is observed and lies at between 75° and 86°. This indicates that the majority of the water molecules are oriented parallel to the surface of matrix regardless of $c_{{{\text{Nacl}}}}$. However, the peak of the angle gradually widens with the addition of salt ions as the concentration of NaCl is below the critical one (${\text{i}}.{\text{e}}.\;c_{{{\text{Nacl}}}} < 0.3\;{\text{mol}}/{\text{L}}$). With the further increasing the salt concentration, the morphological distribution of water molecules is severely restricted (the angular peak are sharper and water molecules are strongly oriented). Indeed, the probability of the $\theta$ and the average permittivity curve are obviously associated since the watershed in the Fig. 4 (blue dotted line) may also be discovered via the $\theta$ distributions. Indeed, the different distributions of $\theta$ implies that the original preorientation of water molecules generated by the graphene surface is gradually bereft with the increase of the concentration of salt solution. As discussed in previously, it can be considered that the augmentation of permittivity with a lower salt concentration is on account of ions that violate the regional orientation of water molecules. This structural and morphological disturbance makes it possible to enhance the degree of freedom of water molecules and results in thus an enhancement in dipole fluctuations. It seems that the induced disorder of water molecules can be related with a favorable entropy production that induced by the increase of permittivity. At a higher $c_{{{\text{Nacl}}}}$, the dipolarized solvent molecules are oriented on the ionic surface (*i.e.* the dielectric saturation phenomenon) and becomes dominant in relation with the entropic contribution. For electrolyte solution in GO channel (see Fig. 7b), the $\theta$ distributions of water molecules are almost superposed in the region of the intermediate NaCl concentration (0.1 mol/L $\le c_{{{\text{Nacl}}}} \le$ 0.4 mol/L). It seems that the superposition of $\theta$ distribution corresponds to the formation of platform of the average permittivity explored in Fig. 4 (red double dot dash line). Nevertheless, this platform is not observed in the case of PG channels and implies a strong competition between the dipolar saturation and the entropic contribution. As shown in Fig. 7b, the profile of $\theta$ becomes sharper gradually as $c_{{{\text{Nacl}}}} >$ 0.4 mol/L and the sharpest peak occurs when the ion concentration reaches 1 mol/L. This latter is well consistent with the average permittivity calculated in Fig. 4, which is even smaller than that of in PG case by the result of the extra compression effect caused by hydroxyl groups insertion on the graphene bilayers, leading to only about half of the dielectric permittivity of salt solution in bulk phase.

As discussed in Part 4, the addition of ions may significantly perturb the hydrogen bonding network of the confined system, and hence leading to a fluctuation of dipolar of water molecules. Table 2 thus present the number of HB bonds per water molecule (${\text{n}}_{{{\text{HB}}}} /{\text{H}}_{2} {\text{O}}$) and the corresponding water dipolar relaxation time ($\tau_{{{\text{HB}}}}$) for confined water and NaCl solution in both PG and GO channels. To this end, we employ the geometrical criteria proposed by Chandra and Chowdhuri^35^ who defined a hydrogen bonds by considering $r_{{{\text{O}} \ldots {\text{H}}}} <$ 2.5 nm and 150° < $\angle_{{{\text{O}} \ldots {\text{H}} - {\text{O}}}}$ < 180°. From this the average life time of HB relaxation are defined by a correlation function of dipole moments described as follow,

9 $${\text{C}}\left( t \right) = \frac{{\left\langle {h\left( {t_{0} } \right)h\left( {t + t_{0} } \right)} \right\rangle }}{{\left\langle {\left( {h\left( {t_{0} } \right)} \right)^{2} } \right\rangle }}$$

where $h\left( {t + t_{0} } \right) = 1$ denotes a specific labelled hydrogen bond intact at time *t* given it is intact at ${\text{t}} = 0$, and is zero else. ${\text{C}}\left( t \right)$ depicts the relaxation of hydrogen bond framework and are not involved in the breaking of hydrogen bond at interval times between zero and t. As shown in Table 2, the number of hydrogen bonds of pure water in GO channel is slightly higher than that of in PG case, which can be ascribed to the existence of OH groups on the GO membrane surface, resulting in the formation of HBs between water molecules and bilayers surface. This is also demonstrated by the slower relaxation time of the dipole moment in GO channel ($\tau_{{{\text{HB}}}} = 2.35\;{\text{ps}}$) than that in PG one ($\tau_{{{\text{HB}}}} = 2.12\;{\text{ps}}$). As the addition of ions in both type of nanochannels the number of HBs and its corresponding dipolar relaxation time decrease. Although the introduction of ions may cause water molecules to orient towards the surface of ions, sufficient water molecules can cope with the effect of a lower number of ions and reorganize themselves to maintain the hydrogen bond network. With the increase of ions concentration (in the range of $0.1\;{\text{mol}}/{\text{L}} < c_{{{\text{Nacl}}}} < 0.5\;{\text{mol}}/{\text{L}}$), the number of HBs in PG channel fluctuates considerably whereas it is almost same in the case of GO channel. The former is due to the formation of a rapid dielectric saturation effect, leading to the orientation of water molecules on the ion surface, and thus the decrease of dielectric permittivity. The latter proves the previous explanation of the permittivity variation, *i.e.* an existence of competition between the dielectric saturation and water-substrate electrostatic interaction. Furthermore, GO bilayers possess a narrower space than PG channel because of the hydroxyl group on the membrane surface, which results in a common fact of a smaller ε in GO channel than that in PG case and is especially salient as $c_{{{\text{Nacl}}}} > 0.4\;{\text{mol}}/{\text{L}}$.

**Table 2:** Number of HB bonds per water molecule and the corresponding water dipolar relaxation time for confined water and NaCl solution in both PG and GO channels.

		PG		GO	
		$\tau_{{{\text{HB}}}} \left( {{\text{ps}}} \right)$	${\text{n}}_{{{\text{HB}}}} /{\text{H}}_{2} {\text{O}}$	$\tau_{{{\text{HB}}}} \left( {{\text{ps}}} \right)$	${\text{n}}_{{{\text{HB}}}} /{\text{H}}_{2} {\text{O}}$
Pure water		2.12 ± 0.04	0.05 ± 0.005	2.35 ± 0.10	0.062 ± 0.005
	0.1	1.72 ± 0.06	0.041 ± 0.004	1.98 ± 0.04	0.052 ± 0.004
	0.2	1.63 ± 0.03	0.039 ± 0.002	1.92 ± 0.02	0.051 ± 0.002
	0.3	2.19 ± 0.05	0.053 ± 0.003	1.93 ± 0.01	0.051 ± 0.002
	0.4	2.35 ± 0.07	0.056 ± 0.002	1.89 ± 0.04	0.05 ± 0.004
$c_{{{\text{Nacl}}}}$(mol/L)	0.5	2.49 ± 0.06	0.06 ± 0.001	2.84 ± 0.11	0.075 ± 0.005
	0.6	2.59 ± 0.03	0.062 ± 0.001	3.03 ± 0.09	0.08 ± 0.002
	0.8	2.7 ± 0.05	0.065 ± 0.001	3.25 ± 0.12	0.086 ± 0.002
	1	2.81 ± 0.04	0.067 ± 0.001	3.41 ± 0.08	0.09 ± 0.003

**Figure 8:**
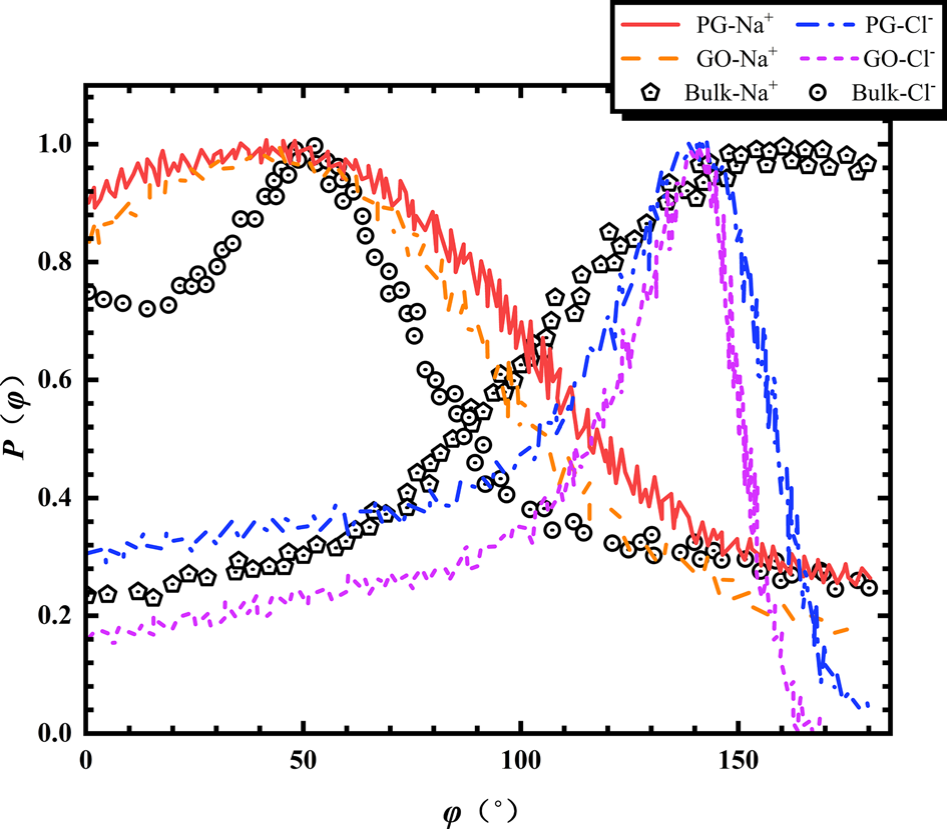
Profile of angle φ of the dipole moment vector of the water molecule and the vector between the ion and the center of mass of the water molecule inside both PG and GO nanochannels.

In summary, the permittivity behavior of salt solutions in both PG and GO bilayers relies on the quantity of ions inside the channel. Indeed, the amount of directional water molecules increases with the arise of the ion concentration, leading to a decrease of ability of dipole fluctuation and thus a decline of the permittivity. Therefore, it is necessary to understand the interaction of water molecules and ions each other under the investigated nanochannels. To this end, we calculate the coordination number by defining a hydration sphere of 0.32 nm to the oxygen of water for Na^+^ and a 0.29 nm to the hydrogen of water for Cl^−^^36^. According to the calculation, both type of nanochannels have almost the same coordination number for Na^+^ and Cl^−^ with 7.8 and 8.9 respectively. This fact is consistent with the findings investigated by Giri et al. who discovered that the coordination number was not affected by the oxidation rate of membrane surface and depends mainly on the interlayer spacing of nanochannel^11^. On the other hand, it should be noted that the coordination numbers of both ions are found identical regardless of the concentration of NaCl solution. As a consequence, the amount of directional water molecules increases progressively with the rise of $c_{{{\text{Nacl}}}}$. In order to analyze quantitatively the orientation of water molecules around ions, we plot in Fig. 8 the profile of angle φ, defined by the dipole moment vector of the water molecule and the vector between the ion and the center of mass of the water molecule, for both types of nanochannels. As expected, a prior orientation of water molecules around Na^+^ and Cl^−^ ions is observed in both bulk and confined phase. As shown in Fig. 8, the peak of φ for both kinds of ions in bulk phase is around 175° for Na^+^ and 50° for Cl^−^ while we find a similar peak of 46° and 136° for Na^+^ and Cl^−^ inside two confined phases, respectively. The different angular peaks for both ions inside the nanochannels with respect to the bulk phase imply a dissimilar structure of water molecules around ions for both phases. For NaCl solution inside the nanochannels, although similar angular distributions have been found for both channels, the curves in GO bilayers are slightly steeper than those in PG channels for both Na^+^ and Cl^−^. This result is attributed to the introduction of hydroxyl groups on the graphene surface, leading to a narrower interlayer space and a stronger confinement effect, and hence a relatively small dielectric permittivity depicted in Fig. 4. Note that chloride ions have been found more susceptible by the confining effect than sodium ions for both confined and bulk phases, which is mainly due to the fact that Cl^−^ have a larger coordination number than Na^+^.

## Conclusion

In the present work, we perform a sequence of MD simulations of aqueous sodium chloride solution confined in a nanochannel formed by either PG or GO sheets with the same interval space. The influence of the concentration of NaCl solution on both PG and GO bilayers has also been investigated.

We observed that the dielectric permittivity in GO channel is smaller than that in PG channel due to the water- substrate electrostatic effect when $c_{{{\text{Nacl}}}} < 0.2$ mol/L. However, this trend is broken when $0.2\;{\text{mol}}/{\text{L}} < c_{{{\text{Nacl}}}} \le 0.4\;{\text{mol}}/{\text{L}}$. Indeed, the increase of salt concentration leads to the precipitous decline of dielectric in PG channel due to the dielectric saturation while a dielectric plateau is formed in GO case, resulting from the competition between the dielectric saturation and water- substrate electrostatic interaction. For higher salt concentrations ($c_{{{\text{Nacl}}}} > 0.4\;{\text{mol}}/{\text{L}}$), although the predominance of dielectric saturation results in a rapid decrease of the permittivity for both PG and GO nanochannels, a relative low dielectric constant in GO channel is presented with respect to that in PG one. This is mainly due to the addition of hydroxyl groups on the membrane surface that compress the space of the channel, and thus limits the rotation of water molecules and leads to the decline of the dielectric permittivity.

The results are provided in prospect of building up a fundamental understanding of micro-level mechanisms in the field of desalination, with the extended target of facilitating the progress of new and urgently needed technologies to cope with the increasing demand for plain water.
